# Wearable System for Biosignal Acquisition and Monitoring Based on Reconfigurable Technologies

**DOI:** 10.3390/s19071590

**Published:** 2019-04-02

**Authors:** Víctor Toral, Antonio García, Francisco J. Romero, Diego P. Morales, Encarnación Castillo, Luis Parrilla, Francisco M. Gómez-Campos, Antonio Morillas, Alejandro Sánchez

**Affiliations:** 1 Department of Electronics and Computer Technology, University of Granada, 18071 Granada, Spain; vtlopez@correo.ugr.es (V.T.); franromero@ugr.es (F.J.R.); diegopm@ugr.es (D.P.M.); encas@ugr.es (E.C.); lparrilla@ditec.ugr.es (L.P.); fmgomez@ugr.es (F.M.G.-C.); 2 Hospital de Alta Resolución de Guadix, 18500 Guadix, Spain; antonio.morillas@ephpo.es; 3 Mando de Adiestramiento y Doctrina, Ejército de Tierra, 18010 Granada, Spain; asanpe9@mde.es

**Keywords:** reconfigurable instrumentation, wearable instruments, ECG, oxygen saturation

## Abstract

Wearable monitoring devices are now a usual commodity in the market, especially for the monitoring of sports and physical activity. However, specialized wearable devices remain an open field for high-risk professionals, such as military personnel, fire and rescue, law enforcement, etc. In this work, a prototype wearable instrument, based on reconfigurable technologies and capable of monitoring electrocardiogram, oxygen saturation, and motion, is presented. This reconfigurable device allows a wide range of applications in conjunction with mobile devices. As a proof-of-concept, the reconfigurable instrument was been integrated into ad hoc glasses, in order to illustrate the non-invasive monitoring of the user. The performance of the presented prototype was validated against a commercial pulse oximeter, while several alternatives for QRS-complex detection were tested. For this type of scenario, clustering-based classification was found to be a very robust option.

## 1. Introduction

Biomedical instrumentation is increasingly relevant, especially in hospital environments, where these instruments are currently a basic tool. Thanks to this type of instrumentation, it is possible to continuously monitor patients and access their historical clinical data. In this context, portable devices present great advantages since they allow this continuous monitoring without requiring the patient to remain hospitalized, unlike clinical instrumentation, which is not easy to transport and requires medical personnel for its configuration and manipulation [1]. The increase rate in the development of this type of devices is steadily growing, as illustrated by the classical Holter monitor, which has now been reduced to a small ear-worn device that monitors several biomedical signals [2]. Therefore, wearable health monitoring systems (WHMS) are increasingly important, while their complexity continues to grow, since a single device has to combine analog circuitry for signal acquisition, advanced digital signal processing, and ever increasing communications capabilities [3]. In addition, the most recent trends in hospital-oriented wearables are the use of new materials and flexible electronics to obtain nanosensors or even implantable sensors to monitor biosignals or chemicals in the body [4,5,6].

This growing interest in health wearables is not only related to hospital and clinical applications. Many commercial devices have appeared in recent years, such as the Apple Watch [7], Samsung’s range of smartwatches [8], and a variety of activity monitors from companies, such as Garmin [9] or Polar [10]. While for most of them the main objective is to keep track of physical and sports activities and user performance, part of their functionality is based on the monitoring of some physiological parameters. In some cases, they are even accurate enough to be used for medical diagnosis under certain conditions, as shown in Reference [11].

One of the challenges for these devices is the growing interest in the monitoring of professionals [12,13], especially in high-risk environments where a health issue during the activity can lead to dangerous situations for both worker and coworkers, such as the case of firemen or military personnel [14]. Any physiological information during this type of situation may help prevent further injuries. In addition, in the case of the military, this information is a really valuable asset for commanders, as they can accordingly manage all available troops and assets. Thus, this work presents a health-monitoring wearable based on reconfigurable technologies. The presented device was aimed at the monitoring of intense activity, such as that of military personnel. The system is capable of simultaneously monitoring electrocardiogram (ECG), oxygen saturation through photoplethysmography (PPG), and user activity, thus providing valuable information about the user’s physical state during deployment.

Several portable systems have been developed to measure biosignals in recent years. Sopic et al. [15] proposed a touch-based system to measure ECG combined with ICG (Impedance CardioGram), in which the objective was early detection of Congestive Heart Failure (CHF). Another example is Reference [16], in which a PPG acquisition system was developed over eyeglasses and used to obtain heart rate (HR) and pulse transist time (PTT), although this system does require an external ECG and a PPG finger-clip. In Reference [17], a preliminary study of eyeglasses for ECG acquisition was presented, comparing the head-acquired ECG with an arm-acquired standard ECG, although no performance analysis was provided. In Reference [18], a wearable system for the acquisition of ECG was presented. It was designed to be integrated into clothing and communicate through an ad hoc 2.4-GHz radio that requires a specific base station. All these proposals presented different solutions for the acquisition of biosignals, but the system presented in this work acquired different biosignals using a single device that did not interfere with user activity. This was achieved through the combination of state-of-the-art signal processing and low-cost reconfigurable technology, thus providing a completely wearable platform for biosignal acquisition and monitoring in hazardous environments. The reconfigurability of the presented system allows other applications to be implemented using the same hardware scheme [19], or to integrate different communication protocols. In this way, the main contributions of this work may be summarized as: integration of ECG, SpO_2_ and motion monitoring into a single device that does not interfere with the activity of the user; ECG acquisition and detection in noisy situations through state-of-the-art digital signal processing; and use of reconfigurable technologies to easily adapt the system to different applications, communication requirements, and scenarios.

The rest of the manuscript is divided into 3 sections. Section 2 is devoted to the materials and methods used in this work, including the description of the proposed system based on reconfigurable technologies. The following section illustrates the performance of the system through experimental results for the different sensing channels of the system. The final section summarizes the main conclusions of this work.

## 2. Materials and Methods

This work presents a prototype wearable instrument based on reconfigurable technologies. As stated above, the main aim of this device was the monitoring of high-risk, high-activity professionals, especially military personnel. For this purpose, the system was created around a Programmable System on Chip (PSoC) 5LP [20], which requires a minimum of external circuitry, but for sensors and electrodes, to manage signal acquisition and processing. Moreover, the inherent reconfigurability of this device allows the implementation of different hardware configurations, enabling a number of applications in this and other fields [19,21,22,23]. In this particular case, the instrument simultaneously monitors ECG, oxygen saturation, and user activity, and can communicate through Bluetooth Low-Energy (BLE) to an external mobile device. The following subsections describe in detail each one of the instrument’s subsystems.

### 2.1. System Overview

**Figure 1:**
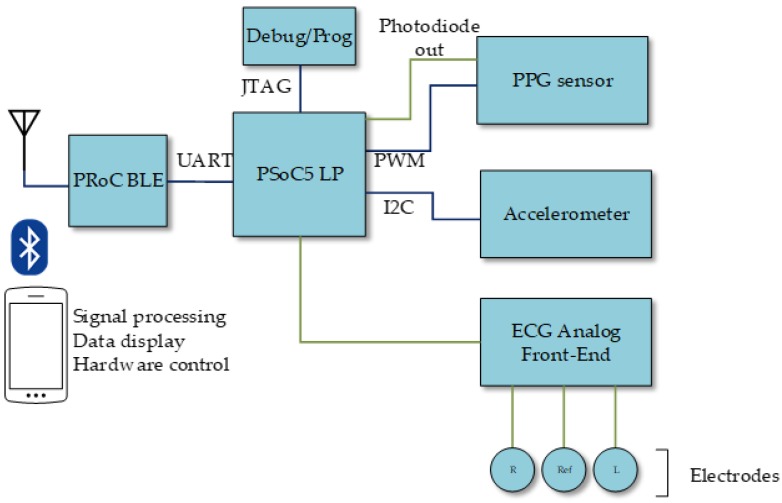
Conceptual schematic of the prototype. PSoC = Programmable System on Chip; BLE = Bluetooth Low-Energy; PPG = photoplethysmography; ECG = electrocardiography; PWM = Pulse Width Modulation; UART = Universal Asynchronous Receiver Transceiver; JTAG = Joint Test Action Group.

Figure 1 shows a conceptual diagram of the presented system. This system was built around a PSoC 5 device, which hosts the main unit, all the control for the different subsystems, and most of the required analog and digital signal processing. The instrument includes three acquisition subsystems:

- Oxygen saturation monitoring: a PPG sensor, controlled by the main unit through a PWM (Pulse Width Modulation) module and its output later processed in the main unit.
- ECG acquisition: an ECG analog front-end for the electrode signals, which are then directly fed to the PSoC 5 for further analog processing, analog-to-digital conversion, and digital processing.
- Motion monitoring: an accelerometer, which communicates via I^2^C (Inter-Integrated Circuit) to the main unit.

Beside those sensing subsystems, the presented instrument also includes:

- Bluetooth module for external communications, managed by the main unit through a UART (Universal Asynchronous Receiver Transceiver).
- Power supply subsystem managing the system battery, recharged through a micro USB connector.
- JTAG (Joint Test Action Group) interface for system programming and debugging.

The reconfigurability of the analog and digital subsystems in the PSoC device allowed efficient implementation of both analog and digital signal datapaths, while also accommodating dedicated digital blocks apart from the microprocessor core. Thus, the PSoC reconfigurable analog cells hosted analog filtering and amplification, as will be detailed later, as well as analog-to-digital conversion. In addition, the reconfigurable digital domain in the PSoC, based in the concept of Universal Digital Block (UDB) [20], can accommodate the UART and I^2^C modules required for communication with the Bluetooth unit and the accelerometer, respectively. These UDBs were also used to implement the PWM module. In any case, the reconfiguration capabilities of this technology enable the adaptation of the system to different application scenarios, such as dynamically changing analog parameters like gain or cut-off frequencies, or the implementation of different communication interfaces. At the same time, reconfiguration makes it possible to use the same hardware in different applications, as in Reference [19], where a very similar hardware configuration, but for the amplifier instrumentation and the different communication interfaces, was used to non-intrusively measure the level inside a tank.

Regarding the ergonomics of the system, initial specifications required the integration of the system into the gear of soldiers (combat helmet, protective glasses, earphones of communication equipment, etc.) or pilots (flight helmet, communication equipment, etc.), since prospective applications were referred to the monitoring of military personnel in different simulation, training, and deployment scenarios. Following the input from military personnel, glasses were found to be the best alternative for this proof-of-concept. Therefore, the entire system, including electrodes, sensors, and battery, should be integrated into the glasses. In this way, a PPG sensor could be placed on the temple of the user. However, it is not possible to place ECG electrodes in this same area, since the artifacts from ocular movement make the signal unsuitable for processing. After careful study, the carotid area in the neck was found to be the best place for ECG acquisition while preserving the wearable nature of the system, with a reference electrode placed on the nasal bridge to take advantage of the frame of the glasses. As will be described below, a 3D-printed proof-of-concept frame was developed to host the system.

The different subsystems are described in detail in the following.

### 2.2. Oxygen Saturation Monitoring

**Figure 2:**
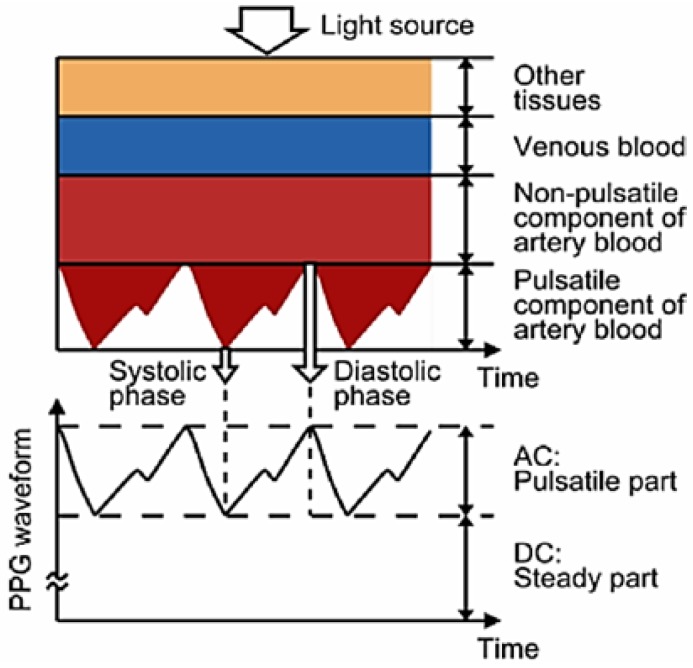
Contributing factors to PPG signals and expected waveform [25].

The presented device measures oxygen saturation through PPG [24]. This technique makes use of the reflection properties of blood in order to obtain the concentration of oxygen in blood. This is possible thanks to the different reflection properties of oxygenated haemoglobin and deoxygenated haemoglobin [25]. These two, which change their relative concentrations during a cycle of the cardiac and pulmonary functions, have a different response to red (R) and infrared light (IR). This makes it possible to determine the level of oxygen saturation in blood, SpO_2_, using a modified version of the Beer–Lambert law [26]:

(1) $${\mathrm{SpO}}_{2}=A+B\frac{V_{AC_{R}}/V_{DC_{R}}}{V_{AC_{IR}}/V_{DC_{IR}}},$$

where *A* and *B* are calibration constants and $V_{AC}$ and $V_{DC}$ are the components of the waveform obtained by the PPG sensor for both R and IR light, as shown in Figure 2.

As it can be deduced from Figure 2, the heart rate (HR) could also be extracted from the PPG signal, since its basic shape is repeated with each heart beat.

**Figure 3:**
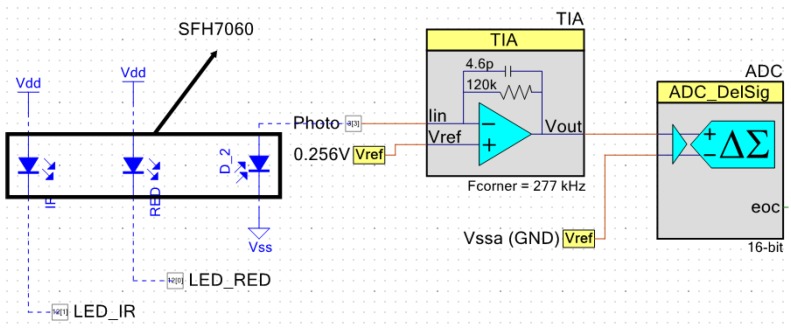
Schematic of the PPG subsystem. TIA = Trans-Impedance Amplifier.

In the presented device, the SFH7060 sensor from OSRAM [27] was used for PPG acquisition. This sensor includes a photodiode and four LEDs: two green, one R, and one IR, although only R and IR LEDs are used. Both the R and IR LEDs, as well as the photodiode, are controlled by the PSoC 5 with a PWM module, which controls the activation time of each LED. The LEDs are thus driven through two digital pins. The output of the photodiode is acquired through a TIA (Trans-Impedance Amplifier), whose gain is controlled adaptively thanks to the reconfigurable nature of the system. This allowed compensation of the sensitivity curve of the sensor. Figure 3 shows the configuration of this subsystem.

The sensor footprint is quite small, only 7.25 × 2.55 mm. It is thus especially suited for these types of wearable applications, even when a development kit version of the sensor has been used for the presented instrument in order to facilitate debugging.

### 2.3. ECG Acquisition

Analysis of the ECG signal is the most widely used diagnostic technique for cardiac diseases. This waveform is measured on the skin and is caused by the electrical response of the different types of cardiac cells during a cardiac cycle. Thanks to this signal, it is possible to detect malfunctions of the heart, which allows the prevention of problems generated by most cardiac conditions [28].

**Figure 4:**
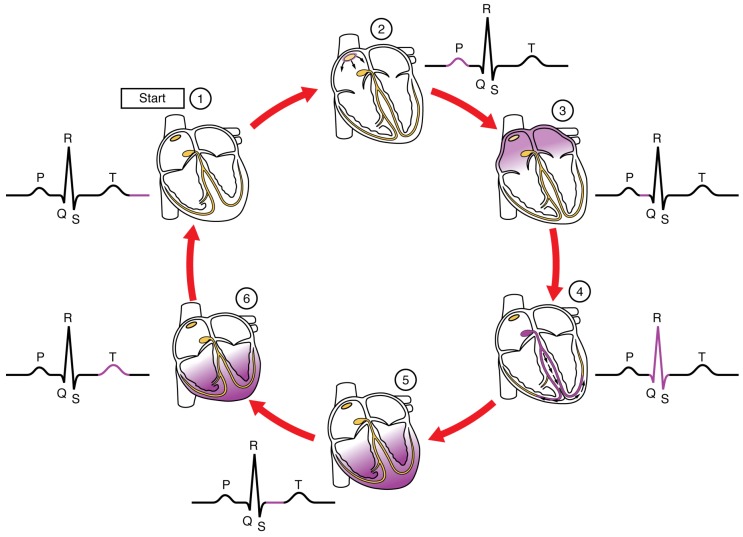
ECG waveform composition [30].

The ECG signal, as shown in Figure 4, is the electrical signal produced on the skin by the cardiac dipole generated during the movement of the heart, which is caused by cyclical electrical depolarization and repolarization of the cells of cardiac tissues [29]. Four important points in the signal are typically named QRST and the times between them are one of the main tools for diagnosis. It is clear that having a good and noise-free ECG signal is essential to draw medical conclusions, thus ECG filtering and processing are key tasks, as discussed in the next subsection.

ECG is usually acquired using electrodes placed in the so-called Einthoven’s triangle [31]. In this way, the three main derivations are obtained depending on the point used as voltage reference. Since the presented device is a prototype for the monitoring of intense activity, the electrodes should be moved to a different position to avoid interference during movement. In this case, the best option for head-mounting both the PPG sensor, placed over the user’s temple, and the ECG electrodes is to place these on the neck just over the carotid arteries, with the ECG reference on the nasal bridge. Thanks to the electrical properties of blood [32], it is thus possible to obtain a clean and strong ECG signal from these carotid electrodes. The electrodes used for this prototype are AMBU Blue Sensor VL, from Ambu, and combine an Ag/AgC sensor with wet gel [33].

**Figure 5:**
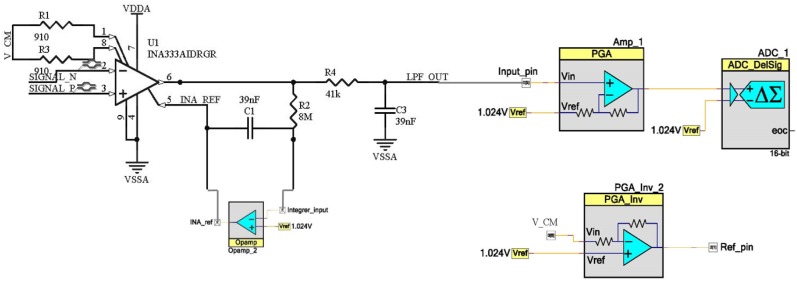
ECG acquisition subsystem.

The ECG acquisition subsystem includes an external INA333 [34] instrumentation amplifier and basic analog filtering, while the rest of the signal datapath is implemented in the PSoC device. Analog filtering is based on two filters, a high-pass filter and a low-pass filter. The high-pass filter is implemented through the reference pin of the instrumentation amplifier [35]. When the low frequency components (below 1 Hz in this case) are introduced in that pin, they will be subtracted from the output signal. In this way, if the low frequency output components are fed back with high gain, it is possible to obtain a high-pass response. To do this, an integrator is used at this pin. As the feedback network needs to amplify the direct current (DC) and low frequency components with high gain, no parallel resistor is placed in the integrator, since limiting DC gain would be a drawback for the filter performance. At the same time, external components are also kept to a minimum. Regarding the low-pass filter, it is a simple resistor–capacitor (RC) network with a cut-off frequency of 100 Hz. The PSoC once again amplifies the acquired signal using a programmable gain amplifier, whose gain can be reconfigured on-the-fly, and finally digitalizes it. Figure 5 shows the schematic of the ECG analog front-end, consisting of PSoC Creator blocks and the external instrumentation amplifier and passive components.

#### 2.3.1. Wavelet-Based ECG Signal Denoising

ECG signals are affected mainly by three types of noise: low frequency noise usually known as baseline wandering, line interferences at 50/60 Hz, and high frequency noise generated by the own electronics and harmonics of the line frequency. Apart from these noise sources, the signal is also affected by artifacts caused by the movement of the subject, while respiration contributes to wandering. The wavelet transform was used in the presented instrument to filter and clean the ECG signals, using the same algorithms presented in Reference [36] that allow simultaneous reduction of high frequency noise and wandering.

The wavelet transform is one of the multi-resolution analysis techniques, which are very useful for signals that vary in time [37] like the ECG. This transform was based on the comparison of the signal to a scaled and time-translated version of a so-called wavelet, which should be adapted to the signal under analysis. In the case of ECG, Daubechies wavelets are usually selected and, in this particular case, the Daubechies wavelet with grade 6, usually known as *db6*, was found to be most appropriate [36].

The discrete version of the wavelet transform, DWT (Discrete Wavelet Transform), is based on successive low-pass and high-pass filters combined with decimation of the signal [38]. At each decomposition level, two vectors are obtained, $d_{n}^{\left(m\right)}$ from the high-pass filter and $a_{n}^{\left(m\right)}$ from the low-pass filter. The vector $d_{n}^{\left(m\right)}$ is known as the detail at *m* level and includes the high frequency information of that decomposition level, while the vector $a_{n}^{\left(m\right)}$ is the corresponding approximation at level *m* and contains low frequency information. Thus, the higher the number of decomposition levels, the better the frequency resolution is, with the low frequency spectrum expanded at each successive decomposition.

It is possible to suppress low frequency noise (wandering) simply by making zero the approximation at the last decomposed level, while high frequency noise can be removed by modifying any values of the detail vectors of the first levels. This modification [36] can be based on either hard or soft thresholding, zeroing any coefficient below a given threshold and subtracting this same threshold from the remaining coefficients for soft thresholding (those remaining coefficients are unaltered with hard thresholding). The minimum number of levels required for proper processing depends on either the sampling frequency as $L=int(log_{2}\left(F_{s}/2\right))$, in case no filters are used, or the cut-off frequency as $L=int(log_{2}\left(F_{max}\right))$ in the other case. Therefore, the number of decomposition levels, the thresholding mode, and the threshold are parameters that can be set in the Android application created for the presented instrument, where the wavelet processing is implemented through 12-coefficient finite impulse response (FIR) filters, as determined by the *db6* family.

#### 2.3.2. QRS Complex Detection and HR Determination

Once the ECG signal has been denoised, it is available for performing any feature extraction or further processing. In the case of HR determination, QRS complexes must be located first. For this purpose, two methods have been used: detection based on thresholds, and a method based on the clustering classification of R peaks [39].

The threshold-based method [36] detects an R peak whenever the signal goes over a certain threshold and its distance to the previous R peak is compatible with several time limits, which are set according to the heart rate. This threshold is usually set to 60% of the signal maximum. This is a simple and effective algorithm, but it is very sensitive to noise and amplitude variations of the signal.

On the other hand, clustering detection [39] is based on the classification of so-called candidate R peaks. These candidate points are local maxima followed by a minimum, which are classified through clustering, using amplitude or time×amplitude as the metric. In the presented case, the classification searches for two groups corresponding to R peaks and noise. Additionally, a correction of FP (false positives) and FN (false negatives) further refines the group of selected R peaks, enabling the calculation of the instantaneous heart rate. Clustering allows a better detection of QRS complexes since it is less affected by the amplitude variation, while the threshold method is limited by the selected threshold. This threshold is specially critical for low signal signal-to-noise ratios (SNRs), thus reducing the accuracy of the system.

### 2.4. Motion Monitoring

The motion monitoring was based on the microelectromechanical systems (MEMS) chip MPU6050 [40], which integrates a 3-axis gyroscope and a 3-axis accelerometer. This chip communicates with the PSoC 5 MPU using an I^2^C port. The data provided by this sensor can be processed for a variety of applications, especially in the fields the prototype is intended for (military use, law enforcement, etc.), and a simple pedometer was implemented to test the functioning of the sensor and debugging its interface. The algorithm is based on the detection of zero crossing, since the shape of the acceleration signal is similar to that of a sinusoid, and each cycle represents a step. In order to refer the acceleration signal to zero, it is adapted using the mean value as reference. Thus, once a reference crossing is detected, the system checks whether the time from the previous detected step is compatible with a valid step or may be caused by other type of movement. For this particular application, any candidate steps that were closer than 0.2 s to the previous one are discarded. In any case, more sophisticated applications can be developed, from a simple alert when the user is stationary for longer than expected to the automatic counting of ammunition based on the detection of weapon recoil, which requires a more refined processing of the acceleration components.

### 2.5. Proof-of-Concept Prototype

**Figure 6:**
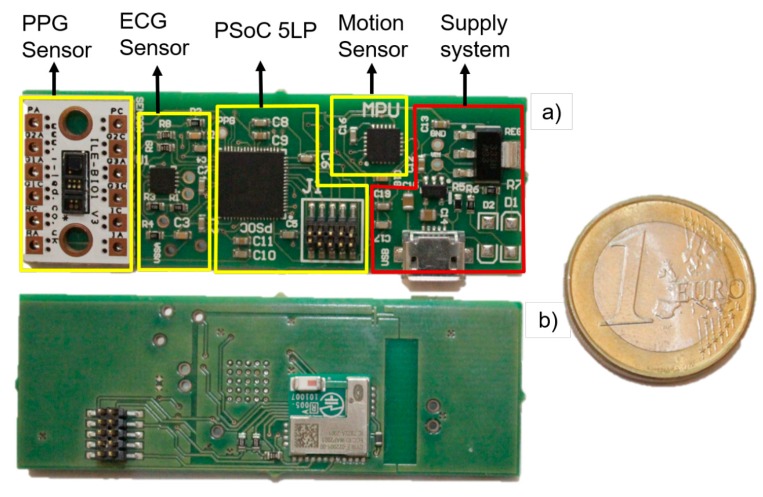
Prototype Circuit Board (PCB) of prototype device: (**a**) top side; (**b**) bottom side with the communications system.

All the subsystems described above have been integrated into a single Prototype Circuit Board (PCB), illustrated in Figure 6. The top-side consists mainly of two sections, one devoted to sensors and the PSoC 5 LP (marked in yellow in Figure 6), and the other one hosting the power subsystem and battery charging through a micro USB connector (marked in red). The bottom side hosts the communication subsystem and ground planes.

#### 2.5.1. Communications Subsystem

Communications were based on BLE. Bluetooth was used in this system because it meets all system requirements in terms of bandwidth and energy consumption. However, alternative communication protocols, such as LoRa, Zigbee, or any other, could be used in this system, depending on the application scenario and providing that the power budget could accommodate them. This change of communication protocols could be easily done thanks to the reconfigurable nature of the system. For this particular application, Bluetooth provides low power consumption while it is widely used in wearable devices. For this, the module PRoC [41] from Cypress Semiconductors was included in the system, which communicates with the main unit through a UART implemented in the PSoC, as described above. Two types of threads are used for communication, depending on whether SpO_2_, ECG, or accelerometer information is sent. When transmitting SpO_2_, heart rate, or step count information, threads are 5 bytes long, each containing the following information:

- **Byte 1:** type of measurement, whether it is heart rate (“*B*”), oxygen saturation (“*O*”), or steps (“*P*”)
- **Byte 2:** hundreds
- **Byte 3:** tens
- **Byte 4:** units
- **Byte 5:** end of thread

**Figure 7:**
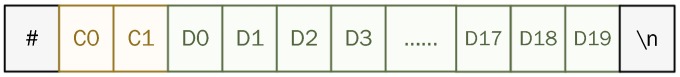
ECG thread format.

In the case of ECG, samples were sent in 24-byte blocks, structured as shown in Figure 7. Within each block, only 20 bytes were data, while the rest were used for the initialization, termination, and codification of the thread. Each signal sample was sent using 2 data bytes, so each thread transmitted 10 ECG samples.

Once these threads were received by the Bluetooth module, they were retransmitted to the mobile device, where the data was decoded and, thus, ready to be used by any apps as required.

#### 2.5.2. Power Subsystem

The power supply system was composed of a small (19 × 15 × 7 mm), flexible 100-mAh Li-ion battery, which provides 3.7 V, a charger, and a regulator. Thus, the battery is charged by a MCP7383 [42], which is fed through a micro USB connector, while an ADP3338 [43] regulator extracts the energy from the battery and provides a 3.3-V, up to 1-A output to the system. This configuration allowed the system to run for longer than 21 h under a normal operation scenario.

#### 2.5.3. Proof-of-Concept Frame and Mobile App

**Figure 8:**
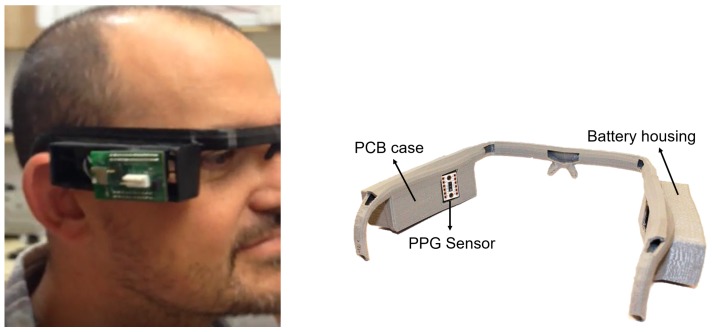
Proof-of-concept frame for prototype integration.

In order to illustrate the possibilities of the presented instrument, a frame replicating the structure of protective glasses was specially designed and 3D-printed. This frame includes two housings, placed on each side of the head, which are used for the battery and for the prototype PCB, respectively. In addition, the interior of the frame is hollow, so all the wiring for electrodes and power supply are contained in the frame, while also allowing the reference electrode to be placed on the nasal bridge. Additionally, this arrangement directly places the PPG sensor on the user’s temple. Figure 8 shows an image of the prototype system in the frame during laboratory tests.

**Figure 9:**
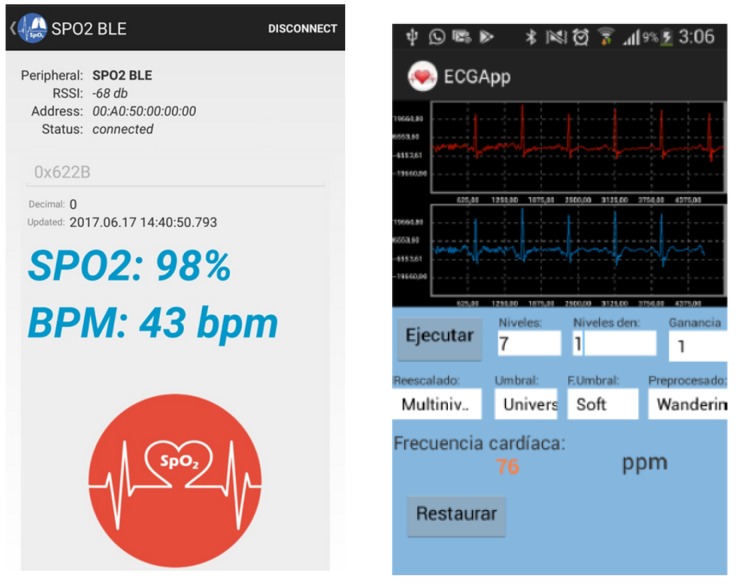
Android app screenshots.

This proof-of-concept was completed with the development of an Android app that acts as graphical user interface (GUI) for the prototype system. This app allows configuration of some hardware parameters, such as the amplifier gain or the activation and deactivation of the Analog to Digital Converter (ADC) in the PSoC 5. It also receives and processes the information gathered by the instrument and can transmit that information, or any data extracted from it, to wherever it is required, thanks to the connectivity capabilities of the hosting mobile device. Figure 9 shows several screenshots of this app.

## 3. Results

The performance of the presented system was evaluated through individual tests of the different sensors included in the system. For this purpose, the performance of the PPG sensing subsystem was compared to that of a commercial pulse oximeter, while ECG acquisition was carried out with different users under different scenarios and evaluated through QRS detection. Finally, as mentioned above, a simple pedometer was implemented to test the functionality of the motion sensor.

### 3.1. PPG Sensor

**Figure 10:**
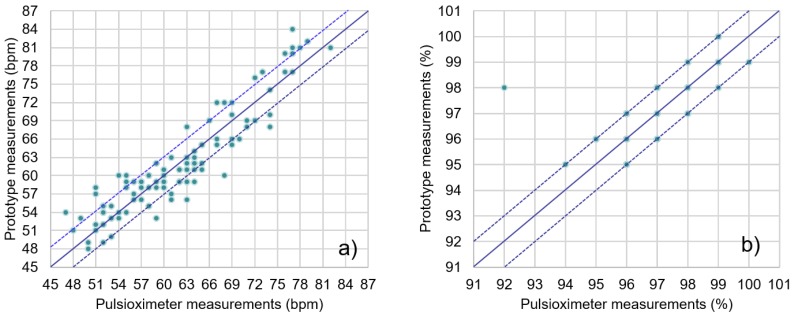
Beats per minute (BPM) (**a**) and SpO_2_ (**b**) results compared to a clinical pulse oximeter (dotted lines represent ±3-BPM and ±1% deviations, respectively).

Data obtained from the system’s PPG sensor were compared to those simultaneously obtained with an off-the-shelf clinical pulse oximeter, the Jumper JPD-500 [44]. This was done with twenty sets of 20-second simultaneous measurements from five different subjects, taking the average SpO_2_ and HR from each instrument for comparison. Results are shown in Figure 10. Most of the readings from the presented system were within the ±3-BPM (beats per minute) range of the pulse oximeter data, with all values in the ±10-BPM range. In the case of oxygen saturation (SpO_2_), all measurements but one were in the ±1% range when compared to the clinical pulse oximeter.

### 3.2. ECG Monitoring

**Figure 11:**
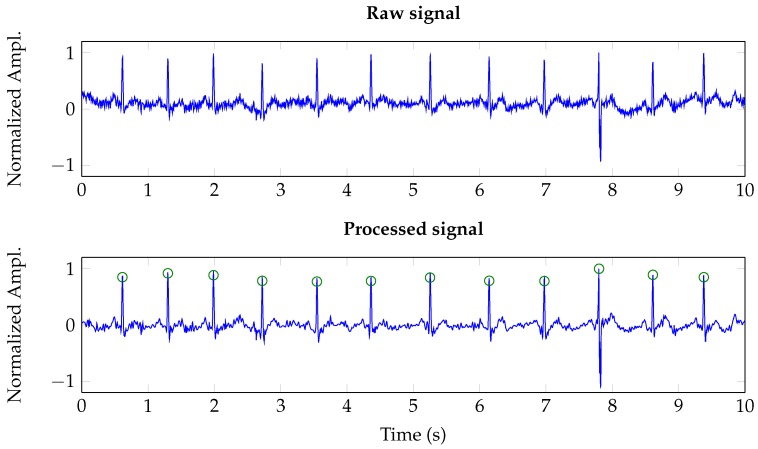
Raw and denoised ECG (dots correspond to detected QRS complexes).

The performance of the ECG acquisition system was analyzed with a QRS complex detection application. For this, the system was tested with seventeen 30-s registers from several subjects. Most of the registers were acquired while the subjects were simulating the usual movements in a flight simulator, as one of the possible applications of the system is the in-cabin monitoring of military pilots. Two additional registers were acquired after the subjects had run up a two-story staircase. Figure 11 shows both a raw signal, i.e., the ADC output, and its wavelet-denoised version. As it is shown, the wavelet-based denoising suppresses wandering and removes high frequency noise. It must be noted that the denoising scheme was tuned to six levels of decomposition and universal soft thresholding [36]. All the registers were manually annotated by a cardiologist and the clinical pulse oximeter was used to validate the HR ranges derived from the analysis of ECG registers.

In order to assess the extraction algorithm, three performance parameters were used: sensitivity, $S_{e}$, positive diagnostic value, PDV, accuracy, $A_{cc}$, and *F*_1_-measure, which are defined as [45,46]:

(2) $$\begin{array}{c} S_{e}=\frac{TD}{TD+FN} \end{array}$$

(3) $$\begin{array}{c} PDV=\frac{TD}{TD+FP} \end{array}$$

(4) $$\begin{array}{c} A_{cc}=\frac{TD}{TD+FP+FN} \end{array}$$

(5) $$\begin{array}{c} F_{1}=2\cdot \frac{PDV\cdot S_{e}}{PDV+S_{e}}=\frac{2TD}{2TD+FN+FP}, \end{array}$$

where TD is the total number of detected QRS complexes, i.e., detected complexes that overlap with manually annotated QRSs; FP is the count of false positives, which correspond to detected QRSs not present in the manual annotations; and FN is the count of false negatives, which are manual annotations that have not been detected by the algorithm. The performance parameters for the different registers are summarized in Table 1 for both threshold-based detection and clustering classification. As shown in Table 1, the threshold-based method was not performing adequately for some subjects, especially those resulting in low amplitude registers, while the overall accuracy was limited to 75.41% due to the inherent limitations of this type of algorithms in noisy scenarios (subjects were simulating the behavior of a pilot, with movement artifacts deteriorating the ECG signal). However, clustering classification improved accuracy up to 94.24% for the same registers, while achieving an almost perfect PDV, since the system tended to lose some QRSs instead of introducing false positives. This proved the efficiency of this approach for noisy scenarios.

### 3.3. Motion Sensor

As stated above, the motion sensor was tested through the implementation of a pedometer. Thus, tests comprised fourteen registers ranging from 20 to 200 steps by different subjects. Table 2 shows the results of the pedometer test. As it can be deduced from the data, the mean error was around 10%, but it must be taken into account that most of this error was derived from the short tests. This was due to the the fact that the initial steps helped calibrate the algorithm, so most of the undetected steps were usually within the first steps of the registers.

### 3.4. Power and Electric Characterization

Table 3 shows power consumption data for the presented system. As stated above, the system includes a 100-mAh battery, so it can operate beyond 2 h 20 min with all modules working simultaneously in non-stop mode. This same battery can supply the system in sleep mode for more than 1200 h. In any case, it must be taken into account that the presented prototype was just a proof-of-concept and was not optimized for energy saving. Moreover, this kind of application scenarios can provide space for larger batteries within the user’s gear.

The presented system requires 74 mW to perform a 30-s ECG recording, or 105 mW to monitor SpO_2_ for 20 s. Thus, it is possible to adapt applications of the presented device to different energy scenarios. For example, the PPG sensor can be used as backup for the determination of HR, since the ECG may be more sensitive to user movement and to noise and interferences and, at the same time, requires more energy (618 μWh vs. 587 μWh). In this way, ECG could be performed only when the user is resting, relying on the motion sensor data, in order to optimize performance and power usage.

## 4. Conclusions

In this work, a low-cost wearable device to monitor ECG, oxygen saturation and activity was presented. The core of the system is a reconfigurable PSoC 5 device, and the instrument was aimed at high-risk, professional applications, such as emergency, law enforcement, or military personnel. The developed system combined different sensors, appropriate signal processing for each sensor subsystem, and Bluetooth communications to achieve a continuous and non-invasive monitoring of the user. A 3D-printed glasses frame was designed to house the instrument as a proof-of-concept for military personnel, while an ad hoc Android app allowed a mobile device to be used as the user interface for the instrument. The reconfigurable nature of the instrument also allowed some of its hardware parameters to be configured from the app, which could also expand the application scope with enhanced external communications and additional processing and computing capabilities in the mobile device.

The combination of reconfigurable hardware with state-of-the-art signal processing makes this proposal a step forward when compared to other wearable solutions. Thus, the possibility of reconfiguration allows a wide range of applications in different fields without redesigning the system. At the same time, the use of cutting-edge algorithms, such as clustering for QRS classification, allowed the design to overcome traditional limitations of wearable systems caused by noisy application scenarios.

## Figures and Tables

**Table 1:** Results for QRS-complex extraction from ECG registers.

Register	Threshold							Clustering						
	TD	FN	FP	$A_{\mathrm{cc}}\%$	PDV %	$S_{e}$%	$F_{1}$%	TD	FN	FP	$A_{\mathrm{cc}}$%	PDV %	$S_{e}$%	$F_{1}$%
1	40	3	1	90.91	97.56	93.02	95.24	41	2	0	95.35	100.00	95.35	97.62
2	5	38	2	11.11	71.43	11.63	20.00	41	2	0	95.35	100.00	95.35	97.62
3	40	3	0	93.02	100.00	93.02	96.39	42	1	0	97.67	100.00	97.67	98.82
4	40	3	0	93.02	100.00	93.02	96.39	43	0	0	100.00	100.00	100.00	100.00
5	1	40	1	2.38	50.00	2.44	4.65	40	0	0	100.00	100.00	100.00	100.00
6	6	35	1	14.29	85.71	14.63	25.00	34	6	2	80.95	94.44	85.00	89.47
7	30	16	4	60.00	88.24	65.22	75.00	34	2	0	94.44	100.00	94.44	97.14
8	29	15	0	65.91	100.00	65.91	79.45	41	3	0	93.18	100.00	93.18	96.47
9	33	6	2	80.49	94.29	84.62	89.19	38	1	1	95.00	97.44	97.44	97.44
10 ${}^{a}$	79	2	0	97.53	100.00	97.53	98.75	77	4	0	95.06	100.00	95.06	97.47
11	33	7	0	82.50	100.00	82.50	90.41	36	4	0	90.00	100.00	90.00	94.74
12	34	6	0	85.00	100.00	85.00	91.89	36	4	0	90.00	100.00	90.00	94.74
13	33	7	0	82.50	100.00	82.50	90.41	36	4	0	90.00	100.00	90.00	94.74
14 ${}^{a}$	68	10	2	85.00	97.14	87.18	91.89	78	0	0	100.00	100.00	100.00	100.00
15	35	3	0	92.11	100.00	92.11	95.89	38	0	0	100.00	100.00	100.00	100.00
16	30	6	0	83.33	100.00	83.33	90.91	36	4	0	90.00	100.00	90.00	94.74
17	34	3	1	89.47	97.14	91.89	94.44	37	0	0	100.00	100.00	100.00	100.00
**TOTAL**	**693**	**212**	**14**	**75.41**	**98.02**	**76.57**	**85.98**	**850**	**47**	**5**	**94.24**	**99.42**	**94.76**	**97.03**

^a^ Subjects had previously run up a two-story staircase.

**Table 2:** Pedometer test results.

Number of Steps	Steps Detected	Error (%)
200	188	6
200	178	11
200	206	3
200	196	2
100	83	17
100	98	2
100	100	0
50	36	28
50	54	8
50	48	4
20	14	30
20	16	20
20	16	20
20	20	0
	**Mean**	**10.78%**

**Table 3:** Power consumption summary for the presented system.

	Consumption (mA)	
Device	Sleep Mode	Active Mode
CPU@24MHz	0.03	9
Bluetooth	0.002	0.21
INA333	0.05	0.05
PSoC modules	0	12
MPU6050	0	0.5
PPG sensor LEDs	0	20
PPG sensor photodiode	0	0.0012
**Total**	**0.082**	**42.7612**
